# Mental and Behavioral Disorders Associated with the Use of Psychoactive Substances and Alcohol: An Epidemiological Analysis in Southern Brazil

**DOI:** 10.1192/j.eurpsy.2024.1734

**Published:** 2024-08-27

**Authors:** L. Bardini, A. Roloff Krüger, G. Moreno Xavier, G. Fiorio Grando, J. Michelon, L. F. Alves Nascimento, J. Adames, A. T. Konzen, G. Pereira Bernd, C. Fontes Augusto, H. Wolmeister, I. Fachinetto Thoen, Y. de França, P. H. Filipin Von Muhlen, F. J. Carvalho da Costa, V. Kayser, P. H. Paesi Dutra, R. Rahal de Albuquerque, T. Garcia Furtado, G. Macelaro, A. C. Castelo, H. Vieira Rodrigues, E. Rockenbach Fidélis, D. Crusius, E. Guidugli, M. F. Valentim de Paula, Y. Marques Loureiro, E. Paiva Borsa, L. de Paula e Souza, G. Ferreira Cruz

**Affiliations:** ^1^Pontifical Catholic University of Rio Grande do Sul, Porto Alegre; ^2^Lutheran University of Brazil, Canoas; ^3^ Federal University of Health Sciences of Porto Alegre; ^4^Federal University of Rio Grande do Sul, Porto Alegre, Brazil

## Abstract

**Introduction:**

Neuropsychiatric disorders are the leading cause of disability worldwide, as seen in cases such as depression, anxiety, bipolar mood disorder and schizophrenia, which can be developed or exacerbated by the use of psychoactive substances. Most mental disorders have an early onset, often leading to early and/or permanent disability, increasing the need and cost of healthcare. Therefore, it is necessary to improve the identification of the epidemiological profile of these cases in the South of Brazil in order to enhance the diagnosis and reduce the costs associated with managing these disorders.

**Objectives:**

The present study aimed to analyze statistical data regarding hospitalizations related to mental disorders caused by the use of psychoactive substances and alcohol in the southern region of Brazil, highlighting the pathological scenario and identifying the most prevalent profiles of these disorders in this region.

**Methods:**

A cross-sectional, descriptive, retrospective, and quantitative study was conducted on hospitalizations of individuals diagnosed with mental and behavioral disorders due to the use of psychoactive substances and alcohol in the states of the Southern region of Brazil (Paraná, Santa Catarina, and Rio Grande do Sul) between February 2020 and December 2022. Data of January 2020 were not available. The data used were collected through the Department of Health Informatics of the Brazilian Unified Health System (DATASUS) in the “Hospital Information System of SUS” section, gathering information regarding the nature of the care, age range, gender, and ethnicity of the patients.

**Results:**

The study covers the years 2020 to 2022, indicating a total of 81,608 hospitalizations, with the year 2022 having the highest number of cases (≈ 37.13%), followed by 2021 (≈ 33.30%) and 2020 (≈ 29.55%). The states with the highest number of hospitalizations were Rio Grande do Sul (≈ 54.90%), Paraná (≈ 29.29%), and Santa Catarina (≈ 15.79%). Urgent hospitalizations accounted for ≈ 87.29% of the total. The most affected age group was 30 to 39 years old (≈ 25.61%). Men were more affected than women (≈ 81.70% and ≈ 18.28%, respectively). Caucasians accounted for ≈ 64.29% of the hospitalizations. The average length of stay was 20.8 days, and the mortality rate was 0.32%.

**Conclusions:**

There is a clear increase in the number of hospitalizations related to mental disorders caused by the use of psychoactive substances in the period from 2020 to 2022 in the southern region of Brazil, with the highest number of cases in the state of Rio Grande do Sul. The most affected population consisted of Caucasian men aged 30 to 39 years old. Furthermore, these results may be related to the increasing trend of psychoactive substance use among the Brazilian population and also the COVID-19 pandemic, which led to a period of underreporting due to social isolation.

**Disclosure of Interest:**

None Declared

